# The efficacy of *Bifidobacterium* quadruple viable tablet in the treatment of diarrhea predominant irritable bowel syndrome: protocol for a randomized, double-blind, placebo-controlled, multicenter trial

**DOI:** 10.1186/s13063-020-04490-0

**Published:** 2020-06-30

**Authors:** Tao Bai, Haoyu Zeng, Yanqin Long, Xiaoqing Li, Xiaohong Sun, Yu Lan, Lingling Gao, Lu Zhang, Zenghui Feng, Xiaohua Hou

**Affiliations:** 1 Division of Gastroenterology, Union Hospital, Tongji Medical College, Huazhong University of Science and Technology, 1277 Jiefang Avenue, Wuhan, Hubei China; 2 Division of Gastroenterology, Sir Run Run Shaw Hospital, Zhejiang University School of Medicine, 3 East Qingchun Road, Hangzhou, Zhejiang China; 3 Division of Gastroenterology, Peking Union Medical College Hospital, No. 1 Shuaifuyuan Wangfujing Dongcheng District, Beijing, China; 4 Division of Gastroenterology, Beijing Jishuitan Hospital, Xicheng District Xinjiekou No. 31 East Street, Beijing, China; 5 Peking University Clinical Research Institute, No.38 Xueyuan Road, Haidian District, Beijing, China; 6 Hangzhou Grand Biologic Pharmaceutical. INC, 63 Jiuhuan Road, Jianggan District, Hangzhou, Zhejiang China

**Keywords:** Irritable bowel syndrome, Diarrhea predominant IBS, Randomized controlled trial, Efficacy, Probiotics

## Abstract

**Background:**

Irritable bowel syndrome (IBS) is one of the most common functional gastrointestinal disorders characterized by recurrent abdominal pain associated with defecation or a change in bowel habits. Leading to significant negative effect on patients’ quality of life and huge financial burden to health system, the management of IBS is a great challenge. Probiotics are considered as an effective therapy; however, in a lack of high-quality evidence of efficacy, no strain- and dose-specific probiotics were recommended in clinical guidelines. This study aims to evaluate the efficacy of the *Bifidobacterium* quadruple viable tablet in the treatment of IBS-D.

**Methods/design:**

A multicenter randomized controlled trial will be performed in fourteen hospitals. A total of three hundred patients who fulfill the eligibility criteria will be stratified divided into an experimental group and a control group randomly in a ratio of 1:1. The experimental group is treated with the *Bifidobacterium* quadruple viable tablet while the control group is treated with placebo. All the patients will receive a 4-week treatment and a 2-week follow-up. The primary outcome is the effectiveness in improving abdominal pain and stool consistency; the secondary outcome includes evaluation of overall symptom relief, frequency of defecation, bloating, urgency of defecation, remedial medication, score of IBS-QOL, and changes of microbiota and metabonomics. Physical examination, vital signs, laboratory tests, adverse events, and concomitant medication will be taken into account for intervention safety assessment during the trial.

**Discussion:**

This multicenter randomized controlled trial may provide high-quality evidence on the efficacy of the *Bifidobacterium* quadruple viable tablet for IBS-D on both physical and mental dimensions in China. To fill the gap of previous probiotic intervention studies, in addition, this study will also present safety assessment which will be a significant emphasis.

**Trial registration:**

ChiCTR1800017721. Registered on 10 August 2018.

## Administrative information

Note: the numbers in curly brackets in this protocol refer to SPIRIT checklist item numbers. The order of the items has been modified to group similar items (see http://www.equator-network.org/reporting-guidelines/spirit-2013-statement-defining-standard-protocol-items-for-clinical-trials/).

**Table:** 

Title {1}	The efficacy of Bifidobacterium quadruple viable tablet in the treatment of diarrhea predominant irritable bowel syndrome: protocol for a randomized, double-blind, placebo-controlled, multicenter trial.
Trial registration {2a and 2b}.	Chinese Clinical Trial Registry, ID: ChiCTR1800017721 (http://www.chictr.org.cn/showproj.aspx?proj=29440). Registered on 10 August 2018. All items from the World Health Organization Trial Registration Data Set could be referred in this entire protocol.
Protocol version {3}	Protocol version No.002 / version date: Nov 22, 2019
Funding {4}	This research is supported by Hangzhou Grand Biologic Pharmaceutical. INC.
Author details {5a}	Tao Bai^1^, Haoyu Zeng^1^, Yanqin Long^2^, Xiaoqing Li^3^, Xiaohong Sun^3^, Yu Lan^4^, Lingling Gao^5^, Lu Zhang^6^, Zenghui Feng^6^, Xiaohua Hou^1, *^ Tao Bai and Haoyu Zeng contributed equally to this work. ^1^Division of Gastroenterology, Union Hospital, Tongji Medical College, Huazhong University of Science and Technology, 1277 Jiefang Avenue, Wuhan, Hubei China. ^2^Division of Gastroenterology, Sir Run Run Shaw Hospital, Zhejiang University School of Medicine. 3 East Qingchun Road, Hangzhou, Zhejiang China. ^3^Division of Gastroenterology, Peking Union Medical College Hospital, No.1 ShuaifuyuanWangfujingDongcheng District, Beijing China. ^4^Division of Gastroenterology, Beijing Jishuitan Hospital, Xicheng District Xinjiekou No. 31 East Street. ^5^Peking University Clinical Research Institute, No.38 Xueyuan Road Haidian District, Beijing China. ^6^Hangzhou Grand Biologic Pharmaceutical. INC, 63 Jiuhuan Road, Jianggan District, Hangzhou, Zhejiang Province.
Name and contact information for the trial sponsor {5b}	XiaohuaHou Address: Union Hospital, Tongji Medical College, Huazhong University of Science and Technology, 1277 Jiefang Avenue, Wuhan, Hubei China. E-mail: houxh@hust.edu.cn
Role of sponsor {5c}	XiaohuaHou is the principal investigator and takes responsibility for the study.

## Introduction

### Background and rationale {6a}

Irritable bowel syndrome (IBS) is one of the most common functional gastrointestinal disorders characterized by recurrent abdominal pain associated with defecation or a change in bowel habits [1, 2]. The prevalence is estimated between 5 and 20% worldwide [3]. Based on the predominant disorder in bowel habits, IBS is classified into four subtypes: constipation predominant IBS (IBS-C), diarrhea predominant IBS (IBS-D), IBS with mixed bowel habits (IBS-M), and unclassified IBS (IBS-U). Among them, IBS-D is the most common one with a proportion of nearly 60% in IBS according to a cross-sectional survey [4]. Although IBS-D affects patients’ quality of life and brings huge financial burden to health system, the management is still a great challenge [5, 6].

Nowadays, more and more studies focus on the role of microbiota in patients with IBS [7–12]. Recently published studies found that compared with normal participants, there is a significant reduction in beneficial bacteria such as *Bifidobacteria* and *Lactobacilli*. Instead, an increase of *Escherichia coli* in patients with IBS-D is observed [11, 12]. Therefore, the change of the microbiota is deemed as an important etiology of IBS-D.

A newly published systematic review supported the benefits of probiotics for patient with IBS, but there was significant heterogeneity as for the various kinds and dose of probiotics [13]. Besides, there are very few high-quality randomized controlled trials with rigorous design [13, 14]. As a result, we need further strong evidence to guide us as for the clinical choice of probiotics.

The *Bifidobacterium* quadruple viable tablet is a new candidate of probiotic product developed and produced by Hangzhou Grand Biologic Pharmaceutical. INC (patent number: ZL01108353.0; International Patent Classification: A61K 35/74). The *Bifidobacterium* quadruple viable tablet is a compound microecological preparation consisting of *Bifidobacterium infantis*, *Lactobacillus acidophilus*, *Enterococcus faecalis*, and *Bacillus cereus*, combining anaerobic with aerobic bacteria. On the one hand, *Bifidobacterium infantis*, *Lactobacillus acidophilus*, and *Enterococcus faecalis* are normal intestinal microbiota inside the intestinal tract of healthy human body. Studies found that direct supplementation of microbiota above can inhibit certain pathogenic bacteria and leads to normal intestinal peristalsis and balance of intestinal microbiome [15–20]. On the other hand, *Bacillus cereus* can colonize in the intestinal tract. They consume oxygen and create an anaerobic environment for anaerobic bacteria to promote the growth and reproduction of anaerobic bacteria such as *Bifidobacteria* [21]. We speculated that the four strains of the *Bifidobacterium* quadruple viable tablet can collaborate to multitarget improvement of the intestinal microenvironment, which indicated a potential therapeutic effect on IBS-D.

### Objectives {7}

The aim of this trial is to evaluate the efficacy of the *Bifidobacterium* quadruple viable tablet in the treatment of IBS-D.

### Trial design {8}

**Fig. 1:**
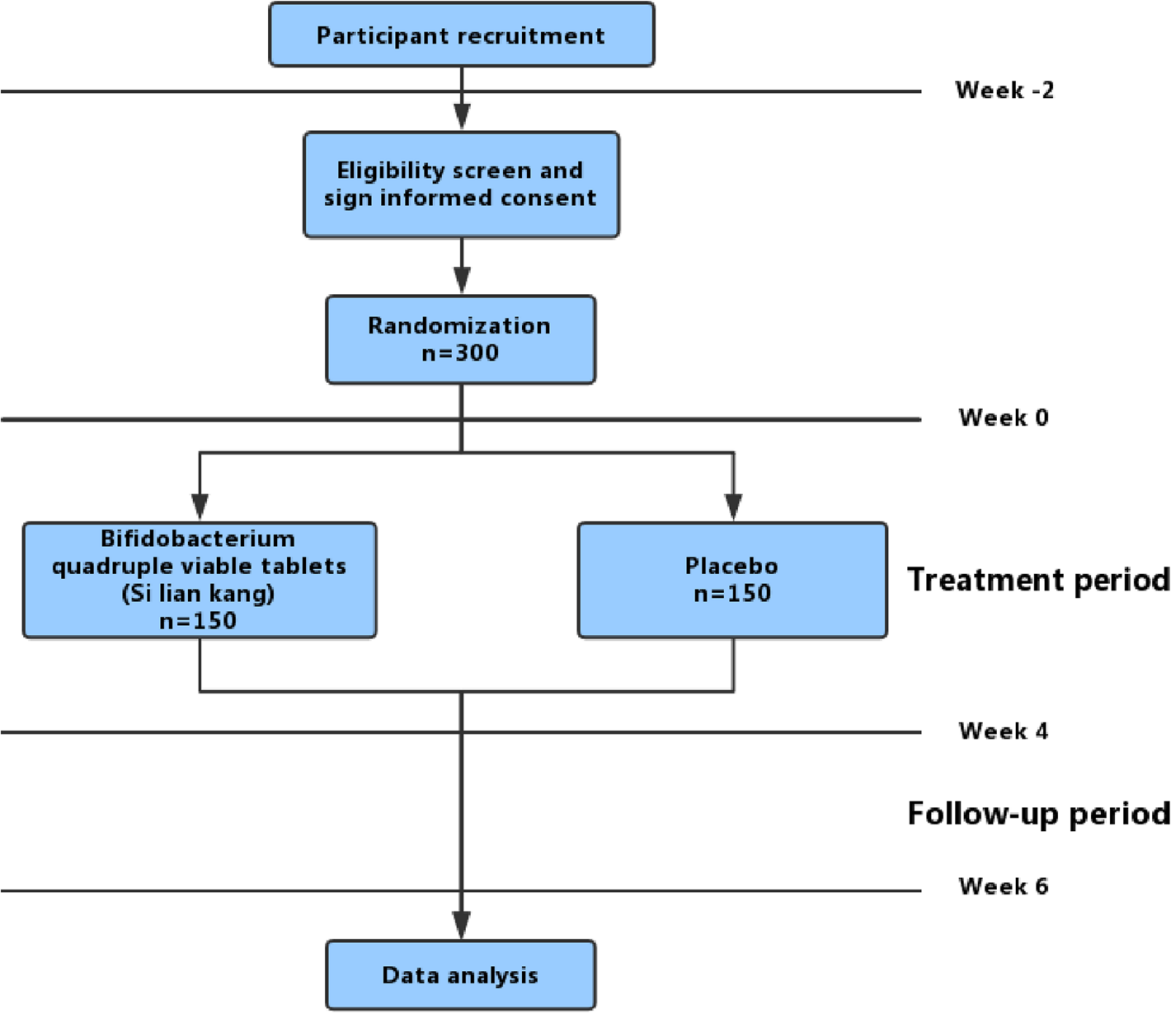
Flowchart of study

**Fig. 2:**
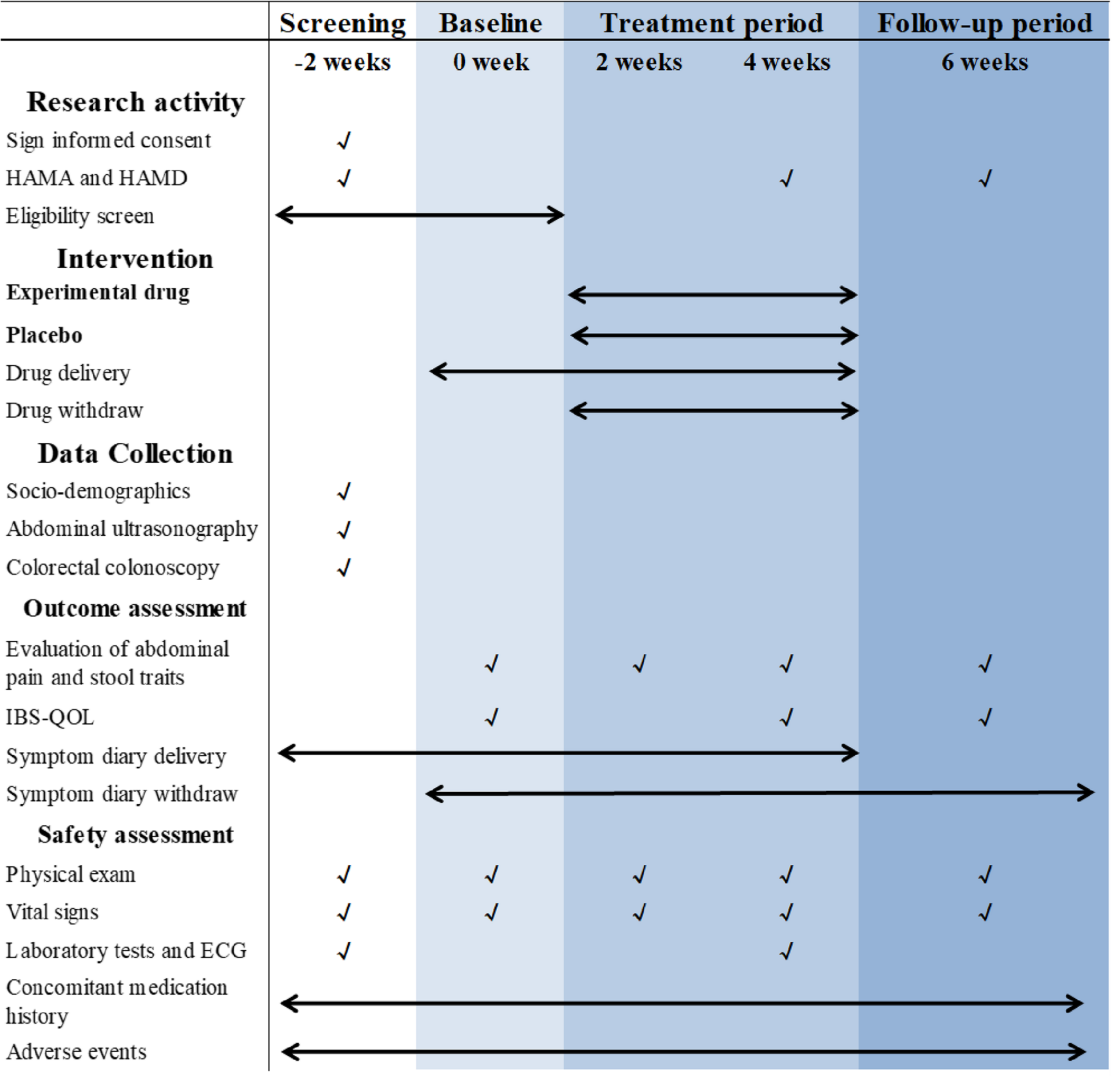
Study schematic diagram. IBS, irritable bowel syndrome; HAMA, Hamilton Anxiety Scale; HAMD, Hamilton Depression Scale; IBS-QOL, IBS-Quality of Life Questionnaire; ECG, electrocardiogram

This study is a parallel, double-blind, placebo-controlled, multicenter randomized controlled trial (RCT). The trial will include three periods: a 2-week screening period, a 4-week treatment period, and a 2-week follow-up period. During the screening period, patients will record a symptom diary every day including abdominal pain intensity, stool consistency, evaluation of symptom remission, and the changes in the pattern of bowel movement. Those who record all items in the symptom diary for at least 10 days during the screening period and fulfill the eligibility criteria will be randomized at a ratio of 1:1 into either the *Bifidobacterium* quadruple viable tablet group or the placebo group. The time schedule and outcome assessment of the trial are presented in Figs. 1 and 2.

The protocol design is based on the Standard Protocol Items: Recommendations for Interventional Trials (SPIRIT) 2013 Checklist (Additional file 1). The overall supervision of our trial will be under the charge of the Clinical Trial Ethics Committee of Huazhong University of Science and Technology; any changes in the protocol will be submitted to and decided by the Ethics Committee. Subjects will receive timely medical treatment when adverse reactions occur during the trial; participants’ health and safety will be the top priority. There will be sufficient time leaving for patients to consider whether to participate in the trial or not.

## Methods: participants, interventions and outcomes

### Study setting {9}

A total of 300 patients diagnosed with IBS-D will be divided randomly into 2 groups (the *Bifidobacterium* quadruple viable tablet group and the placebo group) in a ratio of 1:1. The study is being conducted in the following fourteen hospitals: Wuhan Union Hospital, Peking Union Hospital, Chinese Academy of TCM Wangjing Hospital, Beijing Jishuitan Hospital, The Affiliated Sir Run Run Shaw Hospital of Zhejiang University School of Medicine, Zhejiang Provincial Hospital, Jilin University China-Japan Friendship Hospital, Jilin Provincial People’s Hospital, The First People’s Hospital Of Luoyang, The First People’s Hospital Of Xinxiang, Lianyungang City Hospital, Jingzhou Chinese Hospital, Yan’an University Affiliated Hospital, and The Second Affiliated Hospital of Shandong University of Chinese Medicine.

### Eligibility criteria {10}

#### Inclusion criteria

1. Patients participated voluntarily and signed informed consent.
2. Aged 18–65 years old.
3. Patients were diagnosed as diarrhea predominant irritable bowel syndrome (IBS-D) by Drossman 2016 [22].
4. After 2 weeks of screening (7 days before the beginning of treatment), patients have abdominal pain at least 2 days a week, and the mean value of the most severe abdominal pain was more than 3 (11—NRS report). Meanwhile, the fecal traits were classified as type 6 or 7 at least 2 days.
5. During the screening period (14 ± 3 days before the onset of treatment), the patients completed the symptom diary for at least 10 days.
6. Patients discontinued the drugs used in the pre-screening stage for abdominal symptoms or diarrhea.
7. The lifestyle during the visit (from the first visit before screening to the last visit of the trial) did not make any significant changes in the symptoms of diarrhea and abdominal pain (for example, starting a new diet, or changing the usual exercise pattern).
8. Colonoscopy report within 2 years of tertiary hospitals in China was normal, or the researchers considered no clinical significance (such as chronic colitis diagnosed by congestion and edema, but no erosive ulcer; the diameter of colonic polyps was less than 3 mm, and the number was less than 5).

#### Exclusion criteria

1. Having intestinal organic diseases, such as inflammatory bowel disease, intestinal tuberculosis, malabsorption syndrome, celiac disease, lactose intolerance, or other organic diseases.
2. Having history of abdominal and pelvic surgery, such as cholecystectomy (patients who accepted appendectomy; patients who did not have intestinal complications after cesarean section, such as intestinal obstruction; and patients who accepted endoscopic treatment of intestinal polyps can be included).
3. Having chronic pancreatitis, tumors, peptic ulcers, tuberculous peritonitis, chronic liver disease, liver cirrhosis, and other digestive system non-intestinal diseases (B ultrasonic diagnosis of chronic cholecystitis patients without typical biliary colic can be included).
4. Stool routine results were abnormal: fecal occult blood (+) or red blood cells (+) or white blood cells (+).
5. Having other serious systemic diseases, including serious diseases such as heart, lung, kidney, and other important organ diseases; immunomodulatory diseases; metabolic diseases (diabetes, hyperthyroidism, or hypothyroidism) or malignant tumors; and other diseases such as ovarian cysts and endometriosis.
6. Laboratory tests showed significant abnormalities: (1) hemoglobin < 1 × LLN; (2) serum creatinine was more than 1 × ULN, or creatinine clearance was less than 1 × LLN; (3) abnormal liver function is defined as AST > 1.5 × ULN and/or ALT > 1.5 × ULN and/or total bilirubin > 1.5 × ULN.
7. Patients with severe mental illness or HAMD score > 20 points, or HAMA score > 29 points.
8. Abdominal pain occurs mainly at night during the screening period; patients with unexplained emaciation, fever, jaundice, bloody stool, or black stool; or BMI < 17.
9. Patients with a history of drug abuse or alcohol abuse (the abnormal adaptation caused by alcohol or drug use can lead to clinical morbid state, and at least 1 manifestations in 12 months: ① repeated use of drugs or alcohol to cause negligence or failure in work, academic, or family responsibility, and ② use drugs or alcohol repeatedly on occasions where the body is in danger; ③ legal problems related to the use of drugs or alcohol repeatedly; and ④ although drugs or alcohol causes social or interpersonal problems, they continue to use this substance) and those who are allergic to the drug.
10. During the experiment, the accompanying drugs that affect gastrointestinal motility and function cannot be stopped, such as antibiotics, intestinal microecologic agents, and proton pump inhibitors.
11. Pregnant or breastfeeding women who plan to be pregnant during the trial period.
12. Less than 3 months after attending or completing other clinical trials.
13. Researchers think it is not suitable for the candidates.

### Who will take informed consent? {26a}

Informed consent will be acquired before screening test. All patients recruited will be informed of all contents involved in this trial in oral and written form both by their responsible investigators. There will be sufficient time for patients to consider whether to participate and raise any questions at their concern. And patients who are willing to participate will be required to sign informed consent paper.

### Additional consent provisions for collection and use of participant data and biological specimens {26b}

The investigators must obtain written informed consent for the participation in blood and stool sample collection from each participant before the trial.

### Interventions

#### Explanation for the choice of comparators {6b}

Each *Bifidobacterium* quadruple viable tablet contains a minimum of 0.5 × 106 cfu *Bifidobacterium infantis*, *Lactobacillus acidophilus*, and *Enterococcus faecalis* plus a minimum of 0.5 × 105 cfu *Bacillus cereus*. The placebo tablet contains corn starch without probiotics. The *Bifidobacterium* quadruple viable tablets and the placebo tablets are identical in color, appearance, and packaging. Both of them will be produced and provided by the Hangzhou Grand Biologic Pharmaceutical. INC. (Hangzhou, Zhejiang Province, China).

#### Intervention description {11a}

Subjects of two groups will receive three tablets of either the *Bifidobacterium* quadruple viable tablet or the matched placebo three times a day at 30 min after breakfast, lunch, and dinner, respectively, for consecutive 4 weeks. During the treatment period, researchers will give subjects physical exams and record the results as well as vital signs. Subjects are supposed to keep completing the symptom log every day and record remedial medication and any other drug history permitted in this trial. HAMA, HAMD, IBS-QOL, and laboratory tests will be updated at the end of treatment period.

### Criteria for discontinuing or modifying allocated interventions {11b}

#### Withdrawal criteria

##### Determined by investigator

1. In cases of worsening disease or concomitant complications with a necessity of safety evaluation and curative treatment.
2. With forbidden therapy and medication history during the trial which will lead to bias for efficacy and safety evaluation.

##### Participant request

Patients will be allowed requesting to quit due to their priority, and then, their treatment will abort, regarded as shedding cases and recorded as “treatment failure.” Reasons for withdrawal will be followed and recorded in symptom diary while CRF of every patients will be saved for data analysis,

When subjects with relief of symptoms are unwilling to continue taking the treatment, they need to stop the medication with the researchers’ consent. And the 4-week follow-up will still be conducted after medication as planned.

### Strategies to improve adherence to interventions {11c}

During the trial, experimental drugs and symptom diary will be delivered to patients in the ex-period and returned after the period to ensure the process will be conducted as good as expected. Investigator shall inform patients at the beginning of the trial repeatedly to ensure that they fully understand the importance of timely medication. Patients will be requested to record the code of drugs, date, dose, and time when taking medication in the diary. The diary, leftover medicine, and package will be recycled after the follow-up period, and the tablets will be counted to comprehensively evaluate patients’ compliance to the treatment.

### Relevant concomitant care permitted or prohibited during the trial {11d}

During the trial, any drugs (including antibiotics, parasympathetic inhibitors, muscle relaxants, opiates, other probiotics, antidepressants, anti-anxiety drugs, and Chinese traditional medicine) or other external therapies (including acupuncture, electro-acupuncture, and electric stimulation) that may have impact on the evaluation of the effectiveness of *Bifidobacterium* quadruple viable tablet affecting abdominal pain and diarrhea are not allowed. Drugs that have been used before baseline which are not opposite to the requirement of treatment or forbidden in the exclusion criteria will be permitted during the trial.

### Provisions for post-trial care {30}

There is no anticipated harm and compensation for trial participation.

### Outcomes {12}

#### Primary outcome measures

The primary outcome of this trial is the response rate. A patient is categorized as a responder if the patient is a responder in both pain intensity and stool consistency. The strength of abdominal pain will be evaluated by numerical rating scale using 0 to 10 to represent different levels of pain: 0 means no pain and 10 means severe pain (unable to perform activities of daily living). The consistency of stool will be assessed by using the Bristol Stool Form Scale (BSFS), classified into seven categories:

- Type 1: Separate hard lumps, like nuts (hard to pass)
- Type 2: Sausage-shaped, but lumpy
- Type 3: Like a sausage but with cracks on its surface
- Type 4: Like a sausage or snake, smooth and soft
- Type 5: Soft blobs with clear cut edges (easy to pass)
- Type 6: Fluffy pieces with ragged edges, a mushy stool
- Type 7: Watery, no solid pieces, entirely liquid

The treatment will be considered effective when two criteria are reached simultaneously.

##### Effective in improving abdominal pain for 4 weeks

It will be regarded as remission of abdominal pain for a week when the average of the highest scores of abdominal pain everyday within the week after treatment decreases by more than 30% compared with the week before the treatment. And it will be regarded as the effectiveness of drugs in improving abdominal pain when the remission of abdominal pain lasts for more than half of the treatment period (≥ 2 weeks).

##### Effective in improving stool consistency for 4 weeks

It will be regarded as improvement of stool consistency for a week when the number of days with type 6 or 7 feces within the week after treatment decreases by more than 50% compared with the screening period. And it will be regarded as the effectiveness of drugs in improving stool consistency when the improvement of stool consistency lasts for more than half of the treatment period (≥ 2 weeks).

#### Secondary outcome measures

##### Evaluation of overall symptom relief

Using 1–7 to represents different levels of overall symptom relief at last 7 days:

1. complete remission
2. obvious improvement
3. slightly relief
4. no change
5. slightly worse
6. obvious exacerbation
7. the worst condition imaginable

##### Frequency of defecation

1. once per day
2. twice per day
3. three times per day
4. more than three times per day

##### Bloating (with borborygmus, increasing passing gas, or belching frequently)

1. not at all
2. barely
3. a little
4. moderate
5. rather serious
6. very serious
7. unbearable

##### Urgency of defecation

1. no urgency (feel no need to defecate when it comes to mind)
2. slightly urgent (wish to defecate but not necessarily when it comes to mind)
3. rather urgent (desire to defecate but not immediately)
4. very urgent (have to rush to the bathroom at once)

##### Reduction in BSFS

A reduction of BSFS by three or more points would be adopted as a quantitative indicator to assess the improvement of stool consistency.

##### Use of remedial drugs

Remedial medication will be allowed when subjects’ symptoms aggravate to a health-threatening level evaluated by researchers in both groups. The decision of medication will be at the discretion of clinician for relieving abdominal symptoms and recorded in detail.

##### Quality of life

Subjects will be asked to finish IBS-QOL scale at baseline (week 0), the end of treatment period (week 4), and follow-up period (week 6) successively.

##### Changes of microbiota and metabonomics

Stool samples of subjects will be collected at baseline (week 0) and the end of treatment period (week 4) to study whether changes of microbiota and metabonomics occur after the treatment.

##### Safety evaluation

Subjects will be instructed to report changes of their symptoms truthfully during the trial. Curative effects will be monitored, as well as adverse reactions and unexpected side effects. Adverse events must be recorded in detail whether they are related to the trial or not, including time of occurrence, clinical manifestations, severity, duration, results of laboratory tests, management and outcome, and follow-up times. History of medications is also significant to be on record to analyze causality between adverse events and experimental drugs.

##### Concern on laboratory tests

Electrocardiogram examination and laboratory tests will be performed before and after the treatment for all subjects. Routine blood, urine, stool, stool occult blood, fasting blood glucose, kidney function, and liver function tests will be included. Abnormal changes of results will be on concern and recorded within 24 h after receiving reports; researchers will be supposed to judge whether there are onsets of adverse events. Re-examinations, causality assessments, and necessary intervention will be at the discretion of clinicians.

### Participant timeline {13}

1. Informed consent acquirement
2. Screening period (day − 14 to day 0 ± 3 days)
3. Treatment period (day 1 to day 28 ± 3 days)
4. Follow-up period (day 29 to day 42 ± 3 days)

See Fig. 1.

### Sample size {14}

Sample size calculation was performed by using PASS 13. The 4-week treatment effective rate would be adopted as the main evaluation indicator. According to the systematic review published by Zhang et al. [23], when overall symptom relief is regarded as the main evaluation indicator, the effective rate of probiotics was 50% while placebo was 30% in IBS-intervened RCTs. On this basis and combined with clinical experience, it was assumed that the 4-week treatment effective rate of the experimental group was 50% and that of the placebo group was 30%. In the case of *α* = 0.05 and the power of test was 90% (*β* = 0.10), the required sample size would be at least 124 pairs. Considering the certain shedding and withdrawal samples (52 patients), the planned sample size would be 150 cases/group, and the total would be 300 cases (into a 1:1 ratio).

### Recruitment {15}

In all centers, physicians who participated in this trial were informed of the admittance criteria, and participants will be recruited mainly via physician referrals from the gastroenterology clinics. There are posters and leaflets designed for the recruitment delivering to all centers and placing inside the hospital and community. Those who are willing to participant in this trial will need to contact the investigator and research assistants by telephone.

## Assignment of interventions: allocation

### Sequence generation {16a}

According to the randomization scheme of clinical research, statistical experts randomly code experimental drugs. The experimental drugs are randomly encoded as the unique identifier of the subjects. The subjects and investigators must stay in blind state from the beginning to the end, and changes of schedule will be communicated by the independent staff during the period.

Using the SAS 9.4 statistical software PROC PLAN process to generate a random arrangement of the subjects (the *Bifidobacterium* quadruple viable tablet and placebo) which lists the random coding table, a block randomization model will be applied with a block size of 4.

### Concealment mechanism {16b}

The blinding work will be accomplished by the primary sponsor, principal investigator, and statisticians. Two-dimensional blinding will be conducted. The first dimensional blindness will be implemented for drugs applied in the trial that the *Bifidobacterium* quadruple viable tablet and placebo are in identical appearance and uniformly packaged, while the code of the experimental drug package will be set as the second dimensional blindness. Treatment allocations will be kept into envelops in confidence. One will be saved by staff member from the Clinical Drug Trial Institution, while the others will be sent to each center with drugs as the emergency envelop.

### Implementation {16c}

A statistician not involved in this trial will perform the sequence allocation and block randomization with a block size of 4. The drug number assigned to each center will be random. In this trial, 14 centers are proposed; each center is based on the setting rules, that is, led by the principal investigator and sorted by the initial Chinese phonetic alphabet.

## Assignment of interventions: blinding

### Who will be blinded {17a}

This double-blind controlled trial is designed blinded to the investigators, the subjects, and the outcome assessors.

### Procedure for unblinding if needed {17b}

The blinding will be maintained until serious adverse event occurs. Once blinding is broken, the trial will be aborted and the subject will be marked as a shedding case. The management result will be reported to the supervisor with specific reason, date, and sign recorded on CRF.

## Data collection and management

### Plans for assessment and collection of outcomes {18a}

A training regarding the trial-related procedure will be conducted by the principal investigator for all staff members participating before the implementation of the trial.

In the screening period, data of following contents will be collected:

1. General information including demographic characteristics (gender, age) and previous and present medical history.
2. Scores of Hamilton Anxiety and Depression Rating Scales (HAMA and HAMD) investigated by the trained raters.
3. Physical examination (general and abdominal) and vital signs including temperature, heart rate, respiratory rate, and blood pressure.
4. Laboratory tests including blood routine, urine routine, stool routine, and occult blood test; fasting blood glucose; tests of liver and kidney function; and electrocardiogram.
5. Abdominal ultrasonography and colorectal colonoscopy. Urine pregnancy test only in women of childbearing age.
6. Complete the symptom log everyday including the strength of abdominal pain, fecal traits, level of overall symptom relief, patterns of defecation, remedial medication, and any other drug history permitted in this trial.
7. Adverse events and concomitant medication.

Patients who are admitted in the trial will be allocated randomly into two groups in a ratio of 1:1, heading to the double-blind treatment period.

In the baseline period, investigator shall give subjects physical exam again and subjects will be required to complete the IBS-QOL (quality of life) scale. Data will be recorded in CRF. Feces will be collected to study the microbiome and metabolome at this period.

In the follow-up period, subjects shall update HAMA, HAMD, and IBS-QOL again, and insist to complete the symptom log and records of concomitant drugs and adverse events every day. Researchers shall perform physical exam and record the results and vital signs as same as previous times.

### Plans to promote participant retention and complete follow-up {18b}

Before every follow-up, investigators will make a phone call to patients to ensure if they are ready for the visit. Patients will also be able to contact investigators through the same method when they are confused or in emergency. Once there are patients withdrawing from the trial, the reasons for withdrawal shall be recorded as soon as possible as well as all of the latest outcomes.

### Data management {19}

In this trial, we designed the paper CRF and built the EDC system (Electronic Data Capture System) according to the paper CRF. The investigators were responsible for collecting information from each of the subjects every follow-up, inputting to the EDC system and data reduction. The data will be entered by the investigators or authorized persons (such as CRC), and the data manager will use the EDC system for data management. eCRF will be provided for researchers and authorized persons to record the data.

### Confidentiality {27}

The data manager is responsible for establishing the database which will only open access to investigator and supervisor to keep data and information confidentially. Database will be locked after the blind data verification report and statistical plan are finished; data will not be permitted to be modified anymore.

### Plans for collection, laboratory evaluation, and storage of biological specimens for genetic or molecular analysis in this trial/future use {33}

Stool samples of subjects will be collected at baseline (week 0) and the end of treatment period (week 4). Macrogenomics and metabonomics will be tested to analyze the efficacy of the intervention to the microbiota. Further analysis including the differences between responders and non-responders will be performed.

## Statistical methods

### Statistical methods for primary and secondary outcomes {20a}

#### General analysis

Statistical analysis will be performed by using the SAS 9.4 (or updated version) software (SAS Institute Inc., Cary, NC, USA). In terms of data description, quantitative data will be presented as mean ± standard deviation, median, minimum, maximum, lower quartile (Q1), and upper quartile (Q3), respectively. The categorical data will be presented as frequency and percentage. The comparison of the two groups will be analyzed in proper methods according to the type of indicators. The quantitative data from the two groups will be compared on the basis of data distribution (variance homogeneity, normal distribution) using group *t* test or Wilcoxon’s rank-sum test. A chi-square test or Fisher’s exact test (if the chi-square test is not applicable) will be applied on the categorical data, and Wilcoxon’s rank-sum test or CMH-*χ*^2^ test for the ranked data. Two-sided tests will be applied in all statistical tests, and a *P* value equal to or less than 0.05 will be considered statistically significant (except for special instructions).

#### Interim analyses {21b}

There will be no interim analysis. The study will continue until the 300 participants have completed the study since there were no anticipated problems that are detrimental to the participant.

### Methods for additional analyses (e.g., subgroup analyses) {20b}

The full analysis set (FAS), the per protocol set (PPS), and the safety set (SS) will be included for analysis. The FAS will be obtained on the basis of intention-to-treat (ITT) population, after exclusion from all randomized subjects who receive at least a single-dose treatment through a minimum and rational method. Last-observation-carried-forward will be applied for the estimation of missing data. The PPS is defined as a set including all patients meeting the eligibility criteria; finishing the treatment without major protocol deviations, with good compliance (between 80 and 120%); and completing the CRF as anticipated. The safety set will include those who receive at least a single-dose treatment with data of safety evaluation. Carry-forward of missing data will not be in demand.

### Methods in analysis to handle protocol non-adherence and any statistical methods to handle missing data {20c}

The full analysis set will be obtained on the basis of intention-to-treat (ITT) population, after exclusion from all randomized subjects who receive at least a single-dose treatment through a minimum and rational method. Last-observation-carried-forward will be applied for the estimation of missing data.

### Plans to give access to the full protocol, participant-level data, and statistical code {31c}

The datasets analyzed during the current study are available from the corresponding author on reasonable request.

## Oversight and monitoring

### Composition of the coordinating center and trial steering committee {5d}

The investigators in local centers were responsible for collecting information from each of the subjects every follow-up, inputting to the EDC system and data reduction. The data manager is responsible for establishing the database which will only open access to related investigator and supervisor to keep data and information confidentially. The data will be entered by the investigators in local centers or authorized persons (such as CRC), and the data manager will use the EDC system for data management.

The study will be overseen by the trial steering committee, which comprises the principal investigator, coinvestigators, research coordinator, and study statistician. The trial steering committee will meet before the starting of the trial and every 6 months during the trial. They will be responsible for ensuring that the study is conducted within appropriate and professional ethical guidelines, ensuring the good clinical practice guidelines are observed at all times.

### Composition of the data monitoring committee, its role, and reporting structure {21a}

An independent data monitoring committee comprising experts in clinical epidemiology, statistical staff, gastroenterologist, and clinical research associates (CRAs) is established before the patient enrolment. The trial protocol is reviewed. The data monitoring committee will get a copy of all severe adverse event reports as soon as they become available to the trial investigators. The data monitoring committee will review the reports and report back to the investigators if any further action is required.

There will also be CRAs who are not taking part in this study appointed to be responsible for conducting systematic inspection and monitoring the trial according to the GCP principle, ensuring that the trial scheme is carried out according to the provisions and that the data recorded in the CRF are in accordance with the original data.

### Adverse event reporting and harms {22}

Subjects will be instructed to report changes of their symptoms truthfully during the trial. Curative effects will be monitored, as well as adverse reactions and unexpected side effects. Adverse events must be recorded in detail whether they are related to the trial or not, including time of occurrence, clinical manifestations, severity, duration, results of laboratory tests, management and outcome, and follow-up times. History of medications is also significant to be on record to analyze causality between adverse events and experimental drugs. If any serious adverse event (SAE) occurs, it must be reported to the Province Food and Drug Administration, the State Food and Drug Administration, the primary sponsor institution, the research institutions, and the Ethics Committee within 24 h. Record shall be signed and dated by the researcher who reported the serious adverse event. The blinding will be maintained until SAE occurs. Once blinding is broken, the trial will be aborted and the subject will be marked as a shedding case. The management result will be reported to the supervisor with specific reason, date, and sign recorded on CRF.

When any AE occurs, clinician shall evaluate subject’s condition and provide suitable intervention or treatment. If any SAE occurs, the research institution must take necessary measures to keep subject out of health-threatening danger and stay follow-up until subjects have been resolved.

### Frequency and plans for auditing trial conduct {23}

There will be CRCs who are appointed by the primary sponsor responsible for monitoring every month, checking subjects’ informed consent, screening and inclusion of cases regularly in all research centers, and confirming the following:

1. All eCRFs are filled out correctly and consistent with original data.
2. All miss or omissions of data have been amended with researcher’s sign and date.
3. For each subject, changes of dosage of drugs or therapy, concomitant medication, withdrawal, and omission of examination have been confirmed and recorded.
4. Verify subjects withdrawing or dropping out of trial with explanation recorded on eCRF.
5. Confirm all AEs recorded, and SAEs must be reported and recorded with schedule time.
6. Confirm experimental drugs being supplied, stored, distributed, and withdrew according to the regulation with corresponding records.

### Plans for communicating important protocol amendments to relevant parties (e.g., trial participants, ethical committees) {25}

Once this protocol has been approved by the Ethics Committee, a formal protocol amendment illustration will be required and must be signed by the principal investigator and approved by the Ethics Committee again prior to correction and implementation. The amendment shall be approved and signed by the sponsor after the modification. Any person participating in this trial shall not violate the protocol.

## Dissemination plans {31a}

Dissemination plans include presentations at scientific conferences, scientific publications, stakeholder engagement efforts, and presentation to the public via lay media outlets.

## Discussion

Though probiotics are considered beneficial in IBS treatment according to several meta-analysis and systematical reviews [23–27], specific probiotic recommendations for IBS management are still not possible yet for the lack of data from high-quality RCTs [13, 28–30]. Most previous studies ignore the heterogeneity of IBS and lack of validated symptoms and QOL assessment, which result in obscure outcomes of probiotic effectiveness. This trial aims at demonstrating the efficacy of the *Bifidobacterium* quadruple viable tablet on patients with IBS-D by assessing symptom relief including abdominal pain, stool consistency, bloating, and frequency and urgency of defecation. Non-gastrointestinal health status is also taken into consideration in the form of QOL, psychological status. In comparison with previous studies of probiotic products for IBS treatment, this multicenter collaborative study will have more rigorous methodology and quality assurance.

In addition, having been widely used in clinical practice for a long history, the safety of probiotic preparations is still in concern for the lack of assessment and systematic reporting of adverse events in probiotic intervention studies [31, 32]. To fill the gap in probiotic application, this study also present outcome on safety assessment through monitoring adverse events, comprehensive physical examination, vital signs, biochemical tests, and ECG which reflect patients’ condition objectively. The health of patients is regarded as the priority, and remedial measures are set up for the adverse events. Moreover, changes of microbiota and metabonomics in feces are also taken into consideration; drawing out the connection with symptom relief will be beneficial to gut microbiota researches.

In order to reduce bias, this study adopts adequate sample size and compliance analysis and is conducted in multicenter throughout China to present the outcome in national level. Nevertheless, there are still limitations in this study. As IBS cannot yet be reliably monitored with biomarkers, subjective evaluation scales are adopted as outcome measures, which lack objective evidence and accuracy to verify the efficacy of given treatment. Diet habits vary significantly throughout China. It is difficult to standardize diets for patients from different areas. So diet-related bias may not be controlled easily in such a large-scale study.

As indicated by previous studies, the effectiveness of stain- and dose-specific probiotics remains unclear [28–30], we are expecting this trial will present the efficacy of *Bifidobacterium* quadruple viable tablet in the IBS-D treatment, and the results will be considered as convictive high-quality evidence validating present paradigm.

## Trial status

Participant recruitment is in preparation. The protocol version 2.0 (modified on April 6, 2018) was registered in the Chinese Clinical Trial Registry (ChiCTR1800017721). The recruitment began from July 30, 2018, and was planned to be completed before April 1, 2020.

## Supplementary information

**Additional file 1.** SPIRIT 2013 Checklist: Recommended items to address in a clinical trial protocol and related documents.

## Data Availability

The datasets generated and/or analyzed during the current study are available from the corresponding author on reasonable request.

## Abbreviations

AE: Adverse event
AR: Adverse reaction
BMI: Body mass index
BSFS: Bristol Stool Form Scale
CRA: Clinical research associate
CRC: Clinical research coordinator
CRF: Case report form
ECG: Electrocardiogram
EDC system: Electronic Data Capture System
FAS: Full analysis set
HAMA: Hamilton Anxiety Rating Scale
HAMD: Hamilton Depression Rating Scale
IBS: Irritable bowel syndrome
IBS-D: Diarrhea predominant IBS
IBS-QOL: IBS-Quality of Life Questionnaire
ITT: Intention-to-treat
PPS: The per protocol set
RCT: Randomized control trial
SAE: Serious adverse event
SAR: Serious adverse reaction
SS: The safety set

## References

[1] Longstreth GF, Thompson WG, Chey WD (2006). Functional bowel disorder. Gastroenterology.

[2] Mearin F, Lacy BE, Chang L, et al. Bowel disorders. Gastroenterology. 2016:S0016-5085(16)00222-5 [published online ahead of print, 2016 Feb 18].

[3] Sperber AD, Dumitrascu D, Fukudo S, Gerson C, Ghoshal UC, Gwee KA, Whitehead W (2016). The global prevalence of IBS in adults remains elusive due to the heterogeneity of studies: a Rome Foundation working team literature review. Gut.

[4] Bai T, Xia J, Jiang Y, Cao H, Zhao Y, Zhang L (2017). Comparison of the Rome IV and Rome III criteria for IBS diagnosis: a cross-sectional survey. J Gastroenterol Hepatol.

[5] Wilkins T, Pepitone C, Alex B, Schade RR (2012). Diagnosis and management of IBS in adults. Am Fam Physician.

[6] Rivkin A, Rybalov S (2016). Update on the management of diarrhea-predominant irritable bowel syndrome: focus on rifaximin and eluxadoline. Pharmacotherapy.

[7] Collins SM (2014). A role for the gut microbiota in IBS. Nat Rev Gastroenterol Hepatol.

[8] Bhattarai Y, Muniz Pedrogo DA, Kashyap PC (2017). Irritable bowel syndrome: a gut microbiota-related disorder?. Am J Physiol Gastrointest Liver Physiol.

[9] Shanahan F, Quigley EMM (2014). Manipulation of the microbiota for treatment of IBS and IBD—challenges and controversies. Gastroenterology.

[10] Harris LA, Baffy N (2017). Modulation of the gut microbiota: a focus on treatments for irritable bowel syndrome. Postgrad Med.

[11] Zhuang X, Xiong L, Li L, Li M, Chen M (2017). Alterations of gut microbiota in patients with irritable bowel syndrome: a systematic review and meta-analysis. J Gastroenterol Hepatol.

[12] Dogan B, Belcher-Timme HF, Dogan EI, Jiang Z-D, DuPont HL, Synder N, et al. Evaluation of Escherichia coli pathotypes associated with irritable bowel syndrome. FEMS Microbiol Lett. 2018;365(22). 10.1093/femsle/fny249. 10.1093/femsle/fny249 30299475

[13] McKenzie YA, Thompson J, Gulia P, Lomer MCE (2016). British Dietetic Association systematic review of systematic reviews and evidence-based practice guidelines for the use of probiotics in the management of irritable bowel syndrome in adults (2016 update). J Hum Nutr Diet.

[14] Wilkins T, Sequoia J (2017). Probiotics for gastrointestinal conditions: a summary of the evidence. Am Fam Physician.

[15] Yuan F, Ni H, Asche CV, Kim M, Walayat S, Ren J (2017). Efficacy of Bifidobacterium infantis 35624 in patients with irritable bowel syndrome: a meta-analysis. Curr Med Res Opin.

[16] Lan R, Koo J, Kim I (2016). Effects of Lactobacillus acidophilus supplementation on growth performance, nutrient digestibility, fecal microbial and noxious gas emission in weaning pigs. J Sci Food Agric.

[17] Wang S, Li H, Du C, Liu Q, Yang D, Chen L, Zhu Q, Wang Z (2018). Effects of dietary supplementation with Lactobacillus acidophilus on the performance, intestinal physical barrier function, and the expression of NOD-like receptors in weaned piglets. PeerJ.

[18] Liu C, Zhu Q, Chang J, Yin Q, Song A, Li Z (2017). Effects of Lactobacillus casei and Enterococcus faecalis on growth performance, immune function and gut microbiota of suckling piglets. Arch Anim Nutr.

[19] Samli HE, Dezcan S, Koc F, Ozduven ML, Okur AA, Senkoylu N (2010). Effects of Enterococcus faecium supplementation and floor type on performance, morphology of erythrocytes and intestinal microbiota in broiler chickens. Br Poult Sci.

[20] Duc L, Hong H, Barbosa T, Henriques A, Cutting S (2004). Characterization of Bacillus probiotics available for human use. Appl Environ Microbiol.

[21] Bottone EJ (2010). Bacillus cereus, a volatile human pathogen. Clin Microbiol Rev.

[22] Drossman DA. Functional gastrointestinal disorders: history, pathophysiology, clinical features and Rome IV. Gastroenterology. 2016. 10.1053/j.gastro.2016.02.032. 10.1053/j.gastro.2016.02.032 27144617

[23] Zhang Y, Li L, Guo C, Mu D, Feng B, Zuo X, Li Y (2016). Effects of probiotic type, dose and treatment duration on irritable bowel syndrome diagnosed by Rome III criteria: a meta-analysis. BMC Gastroenterol.

[24] Didari T (2015). Effectiveness of probiotics in irritable bowel syndrome: updated systematic review with meta-analysis. World J Gastroenterol.

[25] Camilleri M (2018). Management options for irritable bowel syndrome. Mayo Clinic Proc.

[26] Barbara G, Cremon C, Azpiroz F (2018). Probiotics in irritable bowel syndrome: where are we?. Neurogastroenterol Motil.

[27] Hungin APS, Mulligan C, Pot B, Whorwell P, Agréus L (2013). Systematic review: probiotics in the management of lower gastrointestinal symptoms in clinical practice - an evidence-based international guide. Aliment Pharmacol Ther.

[28] Rondanelli M, Faliva MA, Perna S, Giacosa A, Peroni G, Castellazzi AM (2017). Using probiotics in clinical practice: where are we now? A review of existing meta-analyses. Gut Microbes.

[29] Mazurak N, Broelz E, Storr M, Enck P (2015). Probiotic therapy of the irritable bowel syndrome: why is the evidence still poor and what can be done about it?. J Neurogastroenterol Motil.

[30] McFarland LV, Evans CT, Goldstein EJC (2018). Strain-specificity and disease-specificity of probiotic efficacy: a systematic review and meta-analysis. Front Med.

[31] Doron S, Snydman DR (2015). Risk and safety of probiotics. Clin Infect Dis.

[32] Wallace TC, MacKay D (2011). The safety of probiotics: considerations following the 2011 U.S. Agency for Health Research and Quality report. J Nutr.

[33] WMA, World Medical Association (2013). Declaration of Helsinki. J Am Med Assoc.

